# A melanosomal two-pore sodium channel regulates pigmentation

**DOI:** 10.1038/srep26570

**Published:** 2016-05-27

**Authors:** Nicholas W. Bellono, Iliana E. Escobar, Elena Oancea

**Affiliations:** 1 Department of Molecular Pharmacology, Physiology, and Biotechnology, Brown University, Providence, RI 02912, USA

## Abstract

Intracellular organelles mediate complex cellular functions that often require ion transport across their membranes. Melanosomes are organelles responsible for the synthesis of the major mammalian pigment melanin. Defects in melanin synthesis result in pigmentation defects, visual deficits, and increased susceptibility to skin and eye cancers. Although genes encoding putative melanosomal ion transporters have been identified as key regulators of melanin synthesis, melanosome ion transport and its contribution to pigmentation remain poorly understood. Here we identify two-pore channel 2 (TPC2) as the first reported melanosomal cation conductance by directly patch-clamping skin and eye melanosomes. TPC2 has been implicated in human pigmentation and melanoma, but the molecular mechanism mediating this function was entirely unknown. We demonstrate that the vesicular signaling lipid phosphatidylinositol bisphosphate PI(3,5)P_2_ modulates TPC2 activity to control melanosomal membrane potential, pH, and regulate pigmentation.

Intracellular ion channels are important for various functions in organelles that underlie critical cellular and organismal physiology, but the identity and function of many organellar ion channels is yet to be discovered. The melanosome is a lysosome-related organelle that produces melanin in pigment cells of the skin and eye. Defects in melanin synthesis and storage result in reduced pigmentation in the skin, hair and eyes, leading to decreased protection against ultraviolet radiation, visual deficits, and increased susceptibility to skin and eye cancers. Although a number of genes encoding putative melanosomal ion transport proteins are essential for pigmentation, ionic signaling in melanosomes remains poorly understood^1^.

Phosphatidylinositol 3,5-bisphosphate [PI(3,5)P_2_] is a low abundance organellar phospholipid (0.1% or less of total cellular phospholipids) that plays an important role in organelle biogenesis and has been implicated as an endolysosomal cation channel agonist^2^,^3^,^4^,^5^. Defects in PI(3,5)P_2_ metabolism result in neurodegenerative disorders and are associated with pigmentation defects. Recently, the direct electrophysiological characterization of several PI(3,5)P_2_-regulated endolysosomal cation channels has uncovered their role in organelle biogenesis, cellular metabolism, and endocytosis^4^,^6^,^7^,^8^,^9^,^10^,^11^.

Two-pore channels (TPC1 and TPC2) are endolysosomal cation channels directly regulated by PI(3,5)P_2_ and implicated in pigmentation^5^,^12^,^13^. The primary function of two-pore channels has been disputed: TPCs were first characterized as nicotinic acid adenine dinucleotide phosphate (NAADP)-activated Ca^2+^ channels in acidic organelles^14^,^15^, while recent electrophysiologal recordings from endolysosomes describes TPCs as Na^+^-selective cation channels^5^,^9^,^11^. As suggested by the conflicting findings, much remains to be learned about the function and regulation of TPCs. TPC2 has been implicated in pigmentation as a determinant of human hair color^12^, a regulator of pigmentation in *Xenopus* oocytes^13^, and a factor in the development of melanoma^16^,^17^, but the mechanisms underlying TPC2-mediated regulation of pigmentation remain unclear.

Here, using direct patch-clamp recordings from skin and eye melanosomes, we report the first melanosomal cation conductance mediated by two-pore channel 2 (TPC2). We show that the organellar signaling lipid PI(3,5)P_2_, an important regulator of pigmentation^3^, activates a TPC2-mediated Na^+^-selective current in melanosomes. We found that TPC2 serves as a negative regulator of pigmentation by increasing melanosomal membrane potential and acidity to decrease cellular melanin content. Thus, in pigment cells, TPC2 mediates a PI(3,5)P2-activated melanosomal Na^+^ channel to regulate pigmentation by modulating the melanosome’s membrane voltage and pH.

## Results

### PI(3,5)P_2_ activates a large inward current in the melanosome (I_PIP2_)

**Figure 1:**
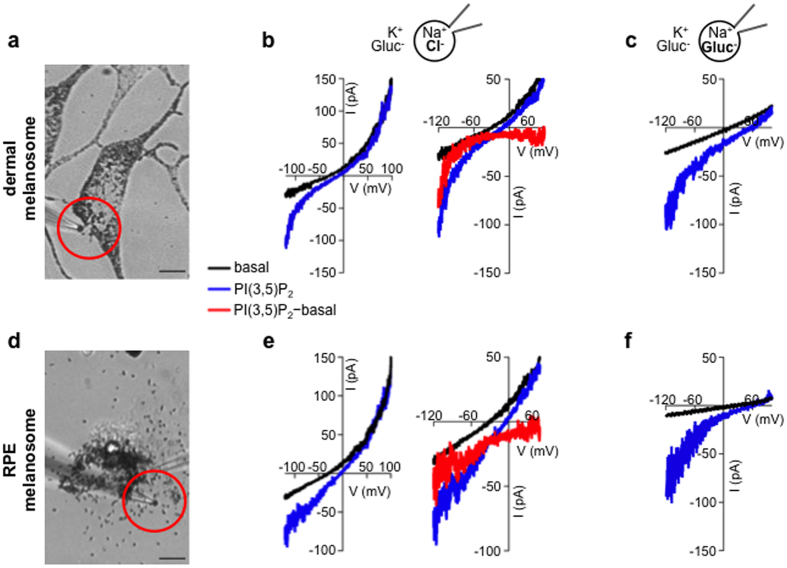
PI(3,5)P_2_ activates an inward current in melanosomes (I_PIP2_). (**a**) Dermal melanosomes dissected from *Oa1*^*−/−*^ melanocytes were patch-clamped using a NaCl-based luminal solution and KGluc-based cytoplasmic solution (scale bar = 10 μm). (**b**) Current-voltage (I-V) relationship from a representative melanosome. In response to voltage ramps, we recorded an outwardly rectifying current with a negative E_rev_ (black). Application of PI(3,5)P_2_ activated an inward current (I_PIP2_) and shifted the E_rev_ in the positive direction (blue). Subtracting the basal current from I_PIP2_ revealed an inward current with a very positive E_rev_ (red) (representative of 7 melanosomes). (**c**) Substituting luminal Cl^−^ for Gluc^−^ reduced the outwardly rectifying basal current amplitude and shifted E_rev_ in the positive direction. Application of PI(3,5)P_2_ activates an inward current similar to I_PIP2_ (representative of 5 melanosomes, p < 0.0001 for the difference in current measured at −120 mV before and after PI(3,5)P_2_). (**d**) Melanosomes dissected from freshly isolated bullfrog RPE were used for patch-clamp experiments (scale bar = 10 μm). (**e**) In a representative RPE melanosome PI(3,5)P_2_ activated an inwardly rectifying current similar to I_PIP2_ measured in dermal melanosomes. The PI(3,5)P_2_ activated component of the current (red) obtained by subtracting the basal current from I_PIP2_ was inward rectifying, with a positive reversal potential (representative of 3 melanosomes). (**f**) Substituting luminal Cl^−^ for Gluc^−^ reduced the outwardly rectifying basal current (black) and revealed the inwardly rectifying current activated by PI(3,5)P_2_ (blue) (representative of 3 melanosomes, p < 0.0001 for the difference in current measured at −120 mV before and after PI(3,5)P_2_).

To investigate endogenous melanosomal ion channels, we measured native currents by direct patch-clamp recordings of melanosomes from a dermal melanocyte cell line derived from mice deficient in ocular albinism 1 (*Oa1*^*−/−*^)^18^, in which melanosomes are enlarged up to 1.5 μm diameter^19^. Melanosomes dissected from *Oa1*^*−/−*^ melanocytes were patch-clamped using a NaCl-based pipette solution to mimic luminal conditions and a K^+^-based bath/cytoplasmic solution containing the impermeant anion gluconate (Gluc^−^) (Fig. 1a). Basal whole-melanosome currents were outwardly rectifying with a negative reversal potential (E_rev_) characteristic of OCA2-mediated melanosomal Cl^−^ currents^20^ (I_basal_). We investigated if melanosomal channel activity could be regulated by PI(3,5)P_2_, an organellar phosphatidylinositol bisphosphate that is important in pigmentation^3^ and activates the endolyosomal cation channels transient receptor potential mucolipin (TRPML)^4^ and two-pore channels (TPCs)^5^. Cytoplasmic treatment with PI(3,5)P_2_ activated a large inward whole-melanosome current and shifted the E_rev_ in the positive direction, but did not significantly affect I_basal_ outward current amplitude (Fig. 1b). To isolate the PI(3,5)P_2_-activated current (I_PIP2_), we subtracted I_basal_ from the total current in the presence of PI(3,5)P_2_ and found a large inward current with a very positive E_rev_ (I_PIP2_) (Fig. 1b). When we minimized Cl^−^-dependent I_basal_ by replacing luminal Cl^−^ with the impermeant anion Gluc^−^, we found that PI(3,5)P_2_ activated a current (I_PIP2_) with similar characteristics revealed by I_basal_ subtraction (Fig. 1c). Furthermore, in melanosomes from melanocytes expressing OCA2 siRNA that have reduced I_basal_, PI(3,5)P_2_ activated a similar inward current (Supplementary Fig. S1a). These results suggest that PI(3,5)P_2_ activates a current distinct from and independent of the outward rectifying OCA2-mediated Cl^−^ current. Because inward I_PIP2_ could result from anions moving into the melanosome but is independent of the major permeant anion Cl^−^, we conclude that it is mediated by cations moving out of the melanosome.

We next tested if the PI(3,5)P_2_-dependent current could also be measured in melanosomes found in the retinal pigment epithelium (RPE) of the eye. By patch-clamping melanosomes from RPE cells of the American bullfrog, *Lithobates catesbeianus* (Fig. 1d), which are larger than those from mammalian RPE cells, we measured a current with properties similar to dermal melanosomal I_PIP2_: PI(3,5)P_2_ activated an inward current that did not affect I_basal_ (Fig. 1e). When basal currents were subtracted from these recorded in the presence of PI(3,5)P_2_, an inward current with a very positive E_rev_ was revealed (I_PIP2_) (Fig. 1e). Substituting luminal Cl^−^ with Gluc^−^ also reduced I_basal_ and isolated I_PIP2_ (Fig. 1f).

### I_PIP2_ has properties similar to endolysosomal TPC2

**Figure 2:**
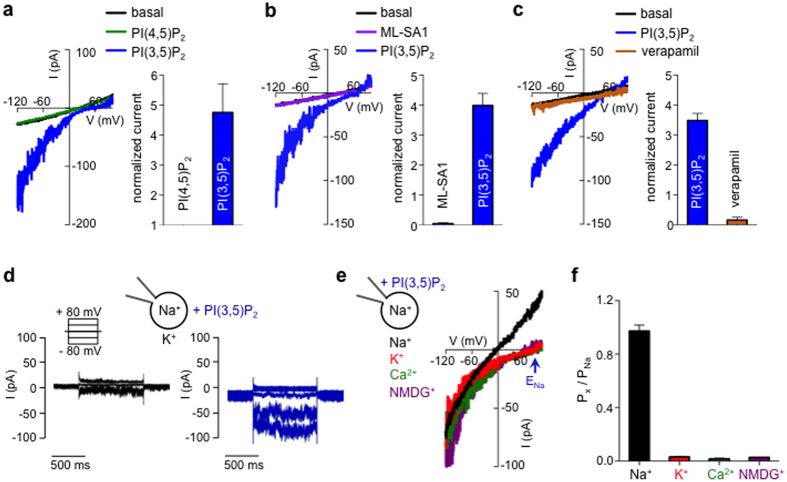
PI(3,5)P_2_ activates a TPC-like current. (**a**) In a representative melanosome from an *Oa1*^*−/−*^ melanocyte, bath application of the intracellular organelle-specific PI(3,5)P_2_ (2 μM), but not the same concentration of plasma membrane PI(4,5)P_2_, activated I_PIP2_. Average PIP_2_-induced increase in current density was measured at −120 mV and normalized to basal currents recorded before PI(4,5)P_2_ or PI(3,5)P_2_ treatment (± s.e.m., n = 3 melanosomes, p < 0.01 for PI(4,5)P_2_- vs. PI(3,5)P_2_-elicited currents). Gluc^−^-based luminal solutions were used to block outwardly rectifying Cl^−^-dependent currents while measuring inward I_PIP2_. (**b**) 2 μM PI(3,5)P_2_ but not the TRPML1 agonist ML-SA1 (25 μM) activated I_PIP2_ in a representative melanosome. Average fold increase in current density measured at −120 mV (± s.e.m., n = 3 melanosomes, p < 0.001 for PI(3,5)P_2_- vs. ML-SA1-elicited currents). (**c**) In a representative melanosome, I_PIP2_ was inhibited by the TPC antagonist verapamil (150 μM). Average fold increase in current density measured at −120 mV (± s.e.m., n = 4 melanosomes, p < 0.0001 for I_PIP2_ vs. I_PIP2_ in the presence of verapamil). (**d**) In a representative melanosome, PI(3,5)P_2_ activated an inward current in response to negative voltage steps; no significant PI(3,5)P_2_-dependent currents were elicited by depolarizing voltages (representative of 3 melanosomes, p < 0.001 for basal currents vs I_PIP2_). (**e**) PI(3,5)P_2_ activated-currents were selective for Na^+^, but not K^+^, Ca^2+^, or the impermeant cation NMDG^+^. Currents were recorded from the same melanosome with 140 mM luminal NaGluc while substituting symmetrical concentrations of cytoplasmic K^+^, NMDG^+^, or 100 mM Ca^2+^ in a Gluc-based solution. (**f**) I_PIP2_ permeability ratios based on E_rev_ measurements (± s.e.m., n = 4 melanosomes, p < 0.0001 for K^+^, NMDG^+^ or Ca^2+^ vs. Na^+^).

To further characterize the properties of I_PIP2_ we used a Gluc^−^-based luminal solution to reduce the contribution of the Cl^−^-dependent outwardly rectifying current (I_basal_). In endolysosomes PI(3,5)P_2_, but not PI(4,5)P_2_, has been shown to activate both TRPML and TPCs^4^,^5^. To test if phosphoinositide regulation of melanosomal I_PIP2_ is specific to the organellar species of PIP_2_, we bath-applied the plasma membrane specific PI(4,5)P_2_ while in whole-melanosome configuration. Basal currents were unaffected by PI(4,5)P_2_ treatment, but, following a wash period, a large inward current was elicited by application of PI(3,5)P_2_ to the same melanosome (Fig. 2a). To test if TRPML channels contribute to the PI(3,5)P_2_-dependent inward current, we used the TRPML-specific agonist ML-SA1^7^. ML-SA1 treatment did not elicit a significant increase in basal currents, while subsequent application of PI(3,5)P_2_ evoked large inward currents in the same melanosomes (Fig. 2b). Additionally, acidifying luminal pH from 6.8 to 4.6, a known regulator of TRPML channels, had no effect on I_PIP2_ (Supplementary Fig. S1c). Treatment with verapamil, however, an antagonist of TPCs^5^,^9^, nearly abolished I_PIP2_ in both dermal (Fig. 2c) and RPE melanosomes (Supplementary Fig. S1b), suggesting that TPCs might contribute to I_PIP2_.

Since two types of endolysosomal TPCs have been characterized, TPC1 and TPC2, we wondered if a specific subtype mediates I_PIP2_. Because TPC1 only is highly sensitive to luminal pH and I_PIP2_ is insensitive to changes in melanosome pH (Supplementary Fig. S1c), I_PIP2_ is not likely mediated by TPC1. Another major difference between the two types of channels is their voltage dependence: TPC1 mediates a depolarization-activated noninactivating conductance while TPC2 is voltage independent, similar to leak channels^11^. Because voltage ramps, as used in our study, mask the voltage-dependence of TPC1 activation, we used voltage steps to test for the presence of voltage-activated currents. Voltage steps between −80 and +80 mV did not elicit channel activation or measureable tail currents in melanosomes, but PI(3,5)P_2_ application activated an inward current (Fig. 2d), suggesting that TPC2 is the most likely melanosomal candidate.

Several reports suggested that TPCs form Ca^2+^ channels^14^,^15^,^21^, while recent evidence suggests they are Na^+^-selective^5^. We determined the cation permeability of I_PIP2_ by measuring E_rev_ with 140 mM Na^+^ in a Gluc^−^-based pipette/luminal solution to reduce Cl^−^-dependent permeation and varying bath/cytoplasmic cations in the presence of cytoplasmic PI(3,5)P_2_. E_rev_ for I_PIP2_ was near 0 mV under symmetrical Na^+^ and >+90 mV for cytoplasmic K^+^, Ca^2+^, or the impermeant cation NMDG^+^, suggesting I_PIP2_ is Na^+^-selective (Fig. 2e). The calculated permeability ratios (P_x_/P_Na_) show that I_PIP2_ is >100 times more selective for Na^+^ than Ca^2+^ and >50 times more selective for Na^+^ than K^+^ (Fig. 2f). Furthermore, currents elicited in symmetrical Na^+^ exhibited leak-like properties, consistent with the lack of voltage-dependent activation. Thus, I_PIP2_ is a highly Na^+^-selective channel with properties similar to TPC2, previously described as a voltage-independent endolysosomal Na^+^ channel^5^,^11^.

### TPC2 localizes to melanosomes to mediate I_PIP2_ and regulate pigmentation

**Figure 3:**
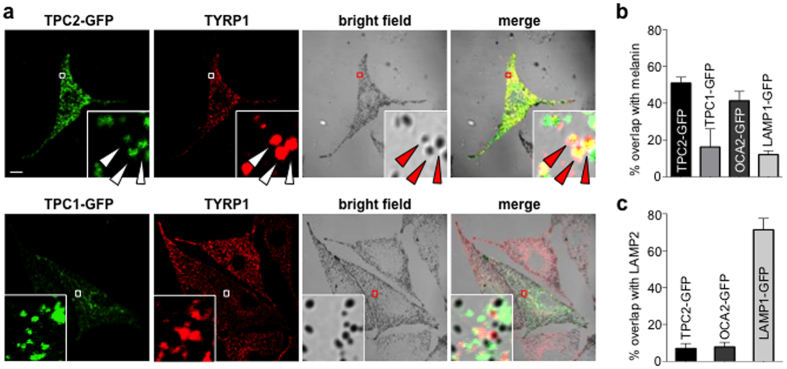
TPC2 localizes to melanosomes in pigment cells. (**a**) GFP-tagged TPC2, but not GFP-TPC1 (green), localized primarily to tyrosinase related protein 1 (TYRP-1, red) and melanin (bright field) containing compartments in melan-a melanocytes. Enlarged images of outlined regions are shown in lower panels. Arrows indicate colocalization of TPC2-GFP and TRYP1 (yellow) and darkly pigmented melanosomes. TPC1 did not significantly overlap with TYRP1 or melanin (scale bar = 10 μm). (**b**) Colocalization analyses of GFP tagged TPC2, TPC1, OCA2 and LAMP1 with melanin-containing melanosomes. TPC2-GFP exhibits significant overlap with melanosomes (50.9 ± 3.2%), similar to the melanosomal protein OCA2-GFP (41.3 ± 5.1%). TPC1 does not significantly overlap with melanosomes (16.2 ± 9.9%), similar to the lysosomal protein LAMP-1 (12.1 ± 2.1%) (n = 13 cells per condition from 3 independent experiments, p < 0.0001 for TPC2 or OCA2 vs. LAMP1, p > 0.5 for TPC1 vs. LAMP1). (**c**) Colocalization analyses of GFP tagged TPC2, OCA2 and LAMP1 with the lysosomal protein LAMP2. Neither TPC2 (7.0 ± 2.7%, p < 0.0001) nor OCA2 (7.9 ± 2.4%, p < 0.0001) exhibit significant overlap with anti-LAMP2 antibodies, while the lysosomal protein LAMP1 shows substantial colocalization with LAMP2 (71.4 ± 6.2%) (n = 4–7 cells per condition from 3 independent experiments).

Because TPCs are regulated by the organellar PI(3,5)P_2_ and have been implicated in pigmentation^5^,^12^, we tested whether they reside in the melanosomal membrane. Expression of GFP-tagged TPC1 or TPC2 in pigment cells (melan-a mouse melanocytes) revealed that only TPC2 overlapped significantly with the melanosomal marker tyrosinase-related protein 1 (TYRP1) and melanin-positive compartments (TPC2 overlap with melanin = 50.9 ± 3.2%, p < 0.0001; TPC1 overlap with melanin = 16.2 ± 9.9%, p > 0.5) (Fig. 3a,b). This result is consistent with the observation that I_PIP2_ has biophysical properties similar to TPC2, which has been implicated in pigmentation. Interestingly, in melanocytes TPC2-GFP did not exhibit significant overlap with the endolyosomal marker LAMP2 (TPC2 overlap with LAMP2 = 7.0 ± 2.7%, p < 0.0001) (Fig. 3c and Supplementary Fig. S2), suggesting that, unlike in other cell types, in pigment cells TPC2 is predominately localized to melanosomes. Although expressed TPC2 preferentially traffics to melanosomes, it is possible that endogenous TPC2 also localizes and functions in the endolysosomes of pigment cells. The GFP-tagged endolysosomal protein LAMP1 colocalized well with LAMP2 (LAMP1 overlap with LAMP2 = 71.4 ± 6.2%, p < 0.0001), but not melanin or TYRP1 (LAMP1 overlap with melanin = 12.1 ± 2.1%, p > 0.5), indicating that not all endolysosomal proteins localize to melanosomes when overexpressed in pigment cells (Fig. 3b,c, Supplementary Fig. S2). Similar to TPC2, the GFP-tagged melanosomal protein OCA2^22^ significantly colocalized with melanin and TYRP1, but not with LAMP2 (OCA2 overlap with melanin = 41.3 ± 5.1%, p < 0.0001; OCA2 overlap with LAMP2 = 7.9 ± 2.5%, p > 0.5), suggesting that in pigment cells TPC2 localization is similar to melanosomal rather than endolysosomal proteins (Fig. 3b,c). Thus, our data suggest that TPC2, previously characterized as an endolysosomal protein, is primarily expressed in melanosomes in pigment cells.

**Figure 4:**
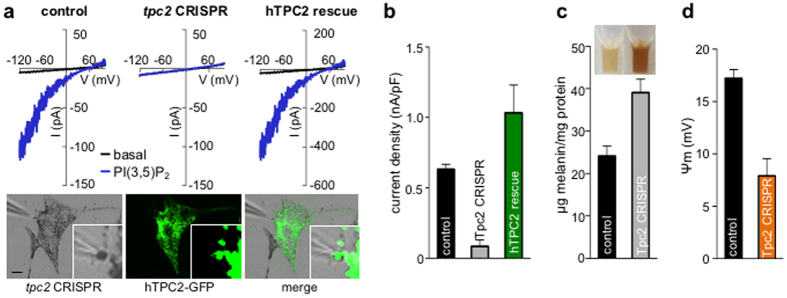
I_PIP2_ is mediated by TPC2. (**a**) Representative I-V relationships of currents recorded before (black) or during (blue) application of 2 μM PI(3,5)P_2_, from melanosomes dissected from control *Oa1*^*−/−*^ melanocytes, *tpc2*-targed CRISPR-Cas9 (*tpc2* CRISPR), or *tpc2* CRISPR rescued with human TPC2-GFP. *Images:* Representative rescue patch-clamp experiment from a melanosome expressing TPC2-GFP dissected from a cell expressing *tpc2*-targed CRISPR-Cas9 (scale bar = 10 μm). (**b**) Average current densities (nA/pF) measured at −120 mV (n = 3–9 melanosomes per condition, p < 0.0001 for *tpc2* CRISPR vs. control or rescue). (**c**) Melanin content was increased in melan-a melanocytes expressing *tpc2*-targeted CRISPR-Cas9 compared with control melanocytes (n = 3 experiments, p < 0.01 for control vs. *tpc2* CRISPR). *Inset:* Solubilized melanin from an equal number of control or *tpc2* CRISPR cells. (**d**) In the presence of 2 μM PI(3,5)P_2_, average resting melanosomal membrane potential (Ψ_m_) was significantly higher in melanosomes from control vs. *tpc2* CRISPR *Oa1*^*−/−*^ melanocytes (n = 3–4 melanosomes per condition, p < 0.01 for control vs. *tpc2* CRISPR).

To determine if TPC2 is required for I_PIP2_, we generated *tpc2*-deficient *Oa1*^*−/−*^ mouse melanocytes using the CRISPR-Cas9 system^23^ (Supplementary Fig. S3a–d). In melanosomes from *Oa1*^*−/−*^ melanocytes expressing *tpc2-*targeted CRISPR-Cas9, PI(3,5)P_2_ failed to activate an inward current (9 out of 13 melanosomes) (Fig. 4a,b). Expression of GFP-tagged human TPC2 in *tpc2-*targeted CRISPR-Cas9-expressing mouse melanocytes was sufficient to rescue I_PIP2_, suggesting that I_PIP2_ is mediated by TPC2 (Fig. 4a,b). Because TPC2 has been previously implicated in pigmentation^12^ and we now find that it functions in melanosomes, we tested if TPC2 expression in melanocytes contributes to pigmentation. We found that reducing the levels of TPC2 by either *tpc2*-targeted CRISPR-Cas9 in melan-a or by siRNA in *Oa1*^*−/−*^ melanocytes had markedly increased cellular melanin content, compared with control melanocytes (Fig. 4c and Supplementary Fig. S3e,f). Thus, TPC2 localizes to melanosomes in pigment cells, where it mediates I_PIP2_ to regulate melanogenesis.

How does TPC2 regulate melanosome function? Because TPC2 forms a Na^+^ leak current, it is likely to contribute to melanosomal membrane potential. To test this, we performed current-clamp recordings of membrane potential in melanosomes from control and *tpc2*-targeted CRISPR-Cas9-expressing *Oa1*^*−/−*^ melanocytes in the presence of PI(3,5)P_2_. In melanosomes from *tpc2*-deficient melanocytes that did not exhibit a PI(3,5)P_2_-induced current the resting membrane potential (Ψ_m_, defined as Ψ_m_ = V_cytosol_ – V_lumen_) was greatly reduced compared with melanosomes from control melanocytes (+7.9 ± 1.7 mV for *tpc2-*deficient and +17.2 ± 0.9 mV for control, Fig. 4d). Thus, TPC2 activity modulates the melanosome’s membrane voltage.

### TPC2 modulates melanosomal pH to regulate pigmentation

The vacuolar proton pump V-ATPase is modulated by membrane voltage and is present in melanosomes where it regulates luminal pH and melanin production^24^,^25^,^26^. Because TPC2 significantly contributes to the membrane potential of melanosomes (p < 0.01 for Ψ_m_ of control vs. *tpc2-*deficient melanocytes in the presence of PI(3,5)P_2_), it is conceivable that TPC2-mediated changes in membrane potential modulate V-ATPase activity and melanosomal pH. To test this hypothesis, we sought to measure melanosomal pH from control and *tpc2*-deficient melanocytes.

**Figure 5:**
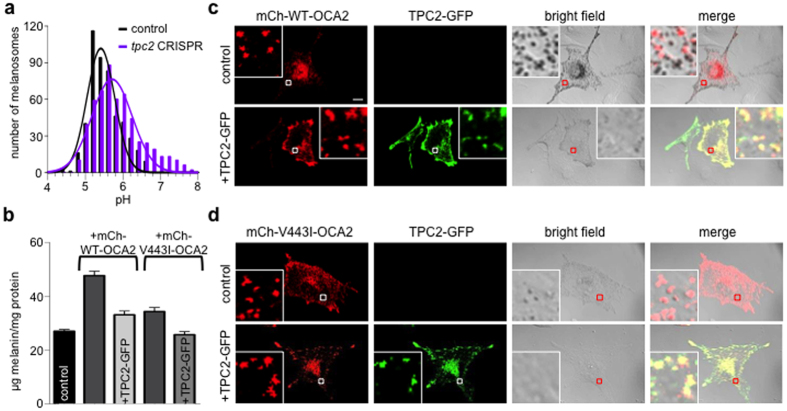
TPC2 regulates melanosomal pH to modulate pigmentation. (**a**) Histogram of pH measurements of melanosomes from B16-F1 melanocytes expressing mCherry (mCh)-tagged V443I OCA2 and control or *tpc2*-targeted CRISPR-Cas9 fitted to Gaussian distributions. n = 496–543 melanosomes from 3 independent experiments, p < 0.0001 for average pH in control (5.51 ± 0.02) vs. *tpc2* CRISPR (5.85 ± 0.03). (**b**) Melanin content (μg melanin/mg protein) of B16-F1 melanocytes (control, 27.0 ± 0.6) was significantly increased by expression of mCh-WT-OCA2 (47.6 ± 3.1, p < 0.001 compared to control) and decreased by coexpressing TPC2-GFP (33.1 ± 1.4, p < 0.01 compared to mCh-WT-OCA2 expressing cells). Expression of mutant mCh-V443I-OCA2 also increased melanin content compared to control B16-F1 melanocytes (34.3 ± 1.5, p ≤ 0.01) and this effect was reversed by coexpressing TPC2-GFP (25.7 ± 1.2, p < 0.01 compared to mCh-V443I-OCA2 expressing cells) (± s.e.m., n = 3 experiments). (**c**) In B16-F1 melanocytes mCherry-tagged wild-type (WT) OCA2 (red) induces pigmentation that is markedly reduced by coexpression with TPC2-GFP (green). Enlarged images of outlined regions shown in lower panels. Scale bar = 10 μm. (**e**) Expression of mCherry-tagged V443I mutant OCA2 (red) in B16-F1 cells induces reduced pigmentation that is further reduced by coexpression with TPC2-GFP (green).

Fluorescence-based pH measurements have not been possible in pigmented melanosomes because fluorescent indicator uptake is impaired and melanin interferes with fluorescence emission. To circumvent these difficulties we used B16-F1 mouse melanocytes, which are weakly pigmented in their basal state. To measure pH in the melanosomes of B16-F1 cells we used the albinism-associated V443I-OCA2 mutant as a melanosomal marker (Supplementary Fig. S4) because it lacks Cl^−^ channel activity, does not significantly increase organellar pH and only modestly induces pigmentation^20^,^27^. These missing features are present with wild-type OCA2 overexpression, which substantially increases pigmentation in B16-F1 melanocytes (Fig. 5b,c), thereby impairing fluorescence-based pH measurements.

Our pH measurements indicate that melanosomes from control B16-F1 melanocytes have a more acidic pH (5.51 ± 0.02) than melanosomes from melanocytes expressing *tpc2*-targeted CRISPR-Cas9 (5.85 ± 0.03) (Fig. 5a). This result is consistent with TPC2’s ability to increase melanosome membrane potential and provide a cation counterflux to support V-ATPase-mediated melanosomal acidification.

Because our results show that TPC2 acidifies melanosomes and melanogenesis is enhanced at more neutral pH values^24^,^25^,^26^, we tested if TPC2 serves as a negative regulator of melanin content by examining its interaction with a positive regulator of melanogenesis, OCA2. Consistent with OCA2’s function as a melanosomal anion channel component that raises organellar pH and enhances melanogenesis^20^, overexpression of OCA2 in B16-F1 melanocytes increased pigmentation by ~75%, as illustrated by the presence of numerous darkly pigmented melanosomes (Fig. 5b,c, top two right panels). We tested if TPC2 negatively regulates OCA2-induced pigmentation by coexpressing mCherry-OCA2 and TPC2-GFP, which colocalized (Fig. 5c, bottom right panels). Interestingly, TPC2-GFP expression reduced OCA2-induced pigmentation by ~30%, indicating that it negatively regulates OCA2-induced melanogenesis (Fig. 5b,c, faint appearance of melanosomes in bottom two right panels). In addition, expression of the albinism-associated mutant V443I-OCA2, which has reduced Cl^−^ transport and pH regulation function^20^, also colocalized with TPC2, but resulted in only a slight increase in B16-F1 pigmentation (~27%) that was completely abolished by coexpression with TPC2-GFP (Fig. 5b,d). Thus, the melanosomal cation channel TPC2 acidifies pH to counterbalance the effect of the OCA2-mediated melanosomal anion channel on luminal pH and to serve as a negative regulator of melanogenesis and pigmentation.

## Discussion

Our results reveal the first cation conductance in melanosomes. We find that in pigment cells the endolysosomal cation channel TPC2 localizes predominantly to melanosomes, where it mediates a Na^+^-selective current to modulate melanosomal membrane potential, pH, and pigmentation. Our findings are consistent with the role of TPC2 in pigmentation as a determinant of human hair color^12^, a factor in the development of melanoma^16^,^17^, and a regulator of pigmentation in *Xenopus* oocytes^13^.

We find that the important organellar signaling molecule PI(3,5)P_2_ regulates the activity of TPC2 in melanosomes. PI(3,5)P_2_ is a known modulator of endolysosomal cation channels^4^,^5^ and affects pigmentation^3^, possibly by regulating melanosome biogenesis^28^. Our results show that PI(3,5)P_2_ is required for the melanosomal TPC2-mediated Na^+^ current; our experimental conditions do not allow us to evaluate whether in intact cells basal PI(3,5)P_2_ is sufficient for TPC2 activity, resulting in a constitutively active Na^+^ current or if an unknown physiological signal modulates PI(3,5)P_2_ levels to regulate melanosomal TPC2 activity.

**Figure 6:**
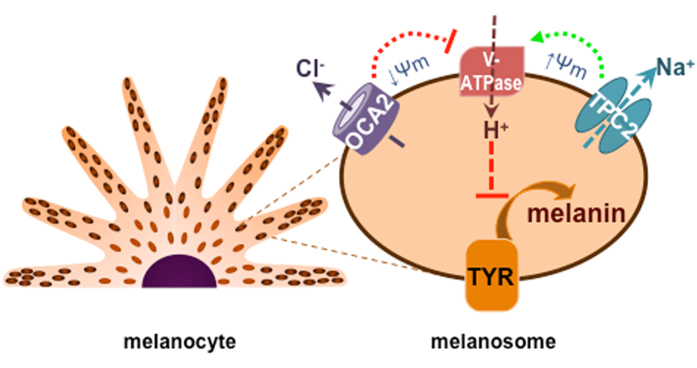
Model of ion channel-mediated regulation of melanosome pH and melanogenesis. TPC2 mediates the major melanosomal Na^+^ conductance to increase melanosome membrane potential (Ψ_m_, Ψ_m_ = V_cytosol_ – V_lumen_) and provide a counter cation to enhance vacuolar ATPase (V-ATPase) activity and increase the acidity of the melanosome lumen, thus reducing the activity of the key melanogenic enzyme tyrosinase (TYR). Additionally, the major melanosomal anion channel mediated by OCA2 expression transports Cl^−^ into the cytoplasm to make the melanosome membrane voltage more negative, thereby decreasing V-ATPase-mediated H^+^ transport and luminal acidity, which enhances the activity tyrosinase and melanin production.

We show that TPC2 functions as a negative regulator of pigmentation by increasing melanosome membrane potential and melanosome acidity, which reduces the activity of the key melanogenic enzyme tyrosinase and subsequent melanogenesis. We hypothesize that TPC2 regulates melanosome pH by providing a cation counterflux to enhance V-ATPase H^+^ transport into the melanosome lumen, consistent with the requirement for an inward cation current in lysosomal acidification^29^. We have recently shown that a melanosomal anion channel mediated by OCA2 reduces organelle acidity to enhance melanogenesis, possibly by decreasing melanosome membrane potential to inhibit V-ATPase activity^20^. We find that the effect of OCA2 on pigmentation is counterbalanced by TPC2, possibly through the regulation of luminal acidity (Fig. 6). It is likely the two conductances regulate one another through voltage-dependent processes and ion homeostasis; it is also possible that the two conductances do not function simultaneously to balance the pH, but rather are differentially regulated by cellular signaling to elicit dynamic changes in melanosomal pH. A more comprehensive model of ionic signaling in melanosomes requires a better understanding of melanosomal membrane potential and luminal ionic concentrations in intact cells; it also entails the molecular identification and characterization of other melanosomal ion channels and transporters. Thus, our studies provide a basis for future work exploring molecular mechanisms underlying ionic signaling in melanosomes and other organelles and could uncover novel therapeutic targets for pigmentation disorders and skin and eye cancers.

## Methods

### Cells and tissue

Immortalized mouse melanocytes melan-a^30^ and melan-*Oa1*^*−/−*^18 were grown in RPMI 1640, 10% fetal bovine serum (FBS, Atlanta Biologicals), 1% penicillin/streptomycin (P/S), 200 nM phorbol 12-myristate-13-acetate (Sigma) at 37 °C and 10% CO_2_. Immortalized melanocytes were obtained from the Wellcome Trust Functional Genomics Cell Bank. B16-F1 melanocytes were grown in DMEM, 10% FBS, and 1% P/S at 37 °C and 5% CO_2_. Transfection of cells was carried out using Lipofectamine 2000 or 3000 (Invitrogen/Life Technologies). Reagents were from Invitrogen/Life Technologies, unless stated otherwise.

#### RPE tissue

RPE tissue was dissected American Bullfrog (*Lithobates catesbeianus*) eyes as previously described 20 and kept at 4 °C for a maximum of 36 hours in a modified Ringer’s solution containing (mM): 111 NaCl, 2.5 KCl, 1 CaCl_2_, 1.5 MgCl_2_, 0.02 EDTA, 3 HEPES, pH 7.6. Bullfrog eyes were obtained as discarded tissue from Dr. Thomas Roberts’ laboratory at Brown University (protocol number 1303990009) and used in agreement with all the ethics rules and regulations.

### Molecular biology

TPC1^9^, TPC2^9^, OCA2 wild type or V443I mutant^20^, and LAMP1 (Addgene plasmid 34831) were tagged with GFP or mCherry.

### CRISPR-Cas9 and siRNA –mediated reduction in protein expression

All-in-one vectors including CMV-driven Cas9 and 20 bp target single-guide RNA sequences (gRNA) were from Genscript. As a control for CRISPR-Cas9 experiments melanocytes expressed Cas9-containing vectors without gene-targeted gRNA.

*tpc2*-targeted gRNA 5′-AGAGCAGCCCCTTCTGGGC-3′ was cloned into the pGS-gRNA-Cas9-Neo vector and expressed using Lipofectamine 2000 (B16-F1 melanocytes) or Lipofectamine 3000 (melan-a and *Oa1*^*−/−*^ melanocytes) following manufacturer’s instructions. Stable cell lines were created using geneticin selection (B16-F1: 1200 μg/ml, melan-a: 1000 μg/ml, *Oa1*^*−/−*^: 1200  μg/ml, melan-a and *Oa1*^*−/−*^ were reduced to 800  μg/ml after 14 days).

#### Analysis of CRISPR-Cas9 edited cell line

Genomic DNA was extracted using a Blood & Cell Culture Mini Kit (Qiagen) using >100,000 cells per sample.

#### Single Clone Sequencing

Genomic DNA was extracted from CRISPR-expressing cells and the CRISPR target sites were amplified with the following primers:

*tpc2 –*F 5′-AGTCAGTCAGTAAGTCCCTCAATCAATCAGT-3′

R 5′-GGATGTTCGGCCACTCACTG-3′;

The PCR products were run on a 1.5% agarose gel and single bands of the predicted size were extracted using QIAquick Gel Extraction Kit (Qiagen). The resulting DNA products were cloned using the TOPO PCR kit (Invitrogen/Life Technologies) according to manufacturer’s protocol. DNA from twenty single cell colonies was extracted and sequenced to screen for gene editing.

#### High Resolution Melt Analysis

50–60 bp amplicons containing CRISPR sites were created using genomic DNA as template and the following primers:

*tpc2 –*F 5′-TGGTGGGATGGCGGCAGA-3′

R 5′-GCCACTGCCTCGGTCCCG-3′

Amplicons were screened for random mutations using real-time PCR (Bio-Rad CFX96) with Precision Melt Supermix (Bio-Rad) according to manufacturer’s instructions.

#### CRISPR-Cas9 Mutation Detection

PCR product was generated from purified genomic DNA using primers described for single clone sequencing to amplify *tpc2* CRISPR sites. Mutation detection was carried out using the Guide-it Mutation Detection Kit (Clontech) according to manufacturer’s protocol. In brief, 800–1000 ng of PCR products were re-annealed to enable heteroduplex formation (95 °C for 5 min, 95 °C to 85 °C ramping at 2 °C/s, 85 °C to 25 °C at 0.1 °C/s, and cooling at 4 °C). Re-annealing products were treated with Guide-it Resolvase for 30 minutes at 37 °C and analyzed on 1.5% agarose gels. The fraction of PCR product cleaved (F_cut_) = (b + c)/(a + b + c) where a = undigested product, b and c = digested products.

Mouse *tpc2*-targeted and control (scrambled) siRNAs were designed with Oligoengine 2.0 software and expressed using the pSUPER RNAi Vector system (Oligoengine). Tpc2-targeted oligos were cloned into pSUPER-GFP-NEO, and expressed in Platinum-E cells to produce retroviral particles. Upon viral transduction of *Oa1*^*−/−*^ mouse melanocytes, stable melanocytes lines expressing *tpc2*-targetd or control siRNA were created using fluorescence-activated cell sorting (FACS) and subsequently kept under G418 selection (800  μg/ml). The relative *tpc2* mRNA levels in melanocytes expressing *tpc2*-targeted or control siRNA were calculated by quantitative PCR relative to actin.

### Melanosomal electrophysiology

#### Melanosome dissection

Enlarged dermal melanosomes were individually dissected from *Oa1*^*−/−*^ mouse melanocytes using a borosilicate patch pipette to cut the cell membrane and push out individual organelles. A new pipette was then used for patch clamp experiments. RPE melanosomes from bullfrog RPE cells were released using two patch pipettes, as previously described^20^.

#### Recording conditions

Melanosome patch-clamp recordings were carried out as previously described^20^. Data were filtered at 2.9 kHz and digitized at 10 kHz using an EPC 10 amplifier (HEKA Instruments) with PatchMaster software (HEKA Instruments). Membrane potentials were corrected for liquid junction potentials. Patch-clamp experiments used borosilicate glass pipettes polished to 10–15 MΩ. Standard voltage-clamp pipette/lumen solution contained (mM): 140 NaCl, 5 KCl, 1 MgCl_2_, 2 CaCl_2_, 10 HEPES, 10 MES, 10 glucose, pH 6.8. Current-clamp pipette/lumen solution contained approximate physiological endolysosomal ion concentrations^9^ (mM): 70 NaCl, 70 KCl, 1 MgCl_2_, 2 CaCl_2_, 10 HEPES, 10 MES, 10 glucose, pH 6.8. Standard bath/cytoplasmic solution contained (mM): 140 K-gluconate, 5 NaCl, 2 MgCl_2_, 0.39 CaCl_2_, 1 EGTA (Ca^2+^ buffered to 100 nM), 10 HEPES, pH 7.2. Current-clamp bath/cytoplasmic solution contained 2 μM PI(3,5)P_2_. Cl^−^ was replaced with Gluc^−^ to block endogenous anion permeability. The holding potential was 0 mV and currents were measured in response to 500 ms voltage ramps from −120 to +100 mV, unless otherwise stated. Whole-organelle current density was calculated by normalizing to capacitance (0.14–0.5 pF). To analyze the effects of some treatments, currents recorded following treatments were normalized to basal currents (normalized current = (I_treatment_ − I_basal_)/I_basal_). Maximal currents were typically recorded ~100 s into treatments and inhibition after ~100 s of treatment. Treatments that did not elicit responses were followed by a wash period and PI(3,5)P2 treatment in the same melanosome.

#### Cation permeability

Relative permeability of I_PIP2_ was determined by measuring E_rev_ after the substitution of bath/cytoplasmic cations from Na^+^ to K^+^, Ca^2+^, or NMDG^+^. Pipette/luminal solution contained 140 mM NaGluc solution and the cytoplasmic/bath solution contained 140 mM X-Gluc, where X = Na^+^, K^+^, or NMDG^+^, or 100 mM CaGluc_2_. Permeability ratios were estimated using the Goldman-Hodgkin-Katz equation: P_X_/P_Na_ = ([Na^+^]_Luminal_/[X]_Cytoplasmic_)(exp(E_rev_F/RT)), P_Ca_/P_Na_ = (4[Ca^2+^]_Luminal_/[Na^+^]_Cytoplasmic_)(exp(E_rev_F/RT)).

### Immunofluorescence microscopy

Melan-a and B16-F1 melanocytes were seeded on coverslips coated with poly-L-lysine (Sigma) and transfected the following day using 4 μg DNA and 3.3 μl of Lipofectamine 2000 for melan-a or 2 μg of DNA and 6 μl of Lipofectamine 2000 for B16-F1. Cells were fixed using 4% paraformaldehyde (Sigma) for 10 min at room temperature, washed with PBS and labeled with primary and secondary antibodies diluted in PBS with 0.2% (w/v) saponin, 0.1% (w/v) bovine serum albumin, 0.02% (w/v) sodium azide. Antibodies used were: mouse anti-TYRP1 (TA99/mel-5, 1:50 dilution, BioLegend/Covance Antibody Products), rat anti-LAMP2 (GL2A7, 1:4, deposited by Granger, B.L. to the Developmental Studies Hybridoma Bank). Goat secondary antibodies conjugated to Alexa 568 or 488 were from Invitrogen. Cells were imaged using a laser-scanning confocal microscope (Zeiss LSM 510) with a 63X objective. Final images were generated from single 0.2 μm stacks and insets magnified using Photoshop software (Adobe). Quantification of colocalization of GFP-tagged proteins and melanin-containing melanosomes or LAMP2 immunostaining was carried out using brightfield and fluorescent confocal images analyzed with ImageJ Fiji software, as previously described^31^. Briefly, after setting an initial threshold the images were made binary for each channel and the area corresponding to fluorescence or melanin-containing melanosomes was quantified. The Image Calculator function was used to multiply the binary images for brightfield melanosomes and fluorescence and the resulting image represents the area of overlap between fluorescently-tagged proteins and melanin-containing melanosomes. The area of overlap of the image resulting from multiplication and the binary image for total fluorescence staining was quantified using the Analyze Particles function to count the area of all structures with an area greater than 5 pixels. The ratio of overlap pixels to total fluorescence pixels gives the % overlap.

### pH imaging

Control or *tpc2*-targeted CRISPR-Cas9-expressing B16-F1 melanocytes transfected one day prior to imaging experiments with mCherry-V443I-OCA2 to identify melanosomes were incubated with 1 μM LysoSensor-ND160 (Invitrogen/Life Technologies) for 5 min. LysoSensor was excited at 405 nm and its emission detected at 417–483 nm (W1) and 490–530 nm (W2) using an Olympus XL fluorescent microscope. The ratio of emissions (W1/W2) in melanosomes expressing mCherry-V443I-OCA2 was assigned to a pH value based on a calibration curve generated with solutions containing 125 mM KCl, 25 mM NaCl, 24 μM Monensin, and varying concentrations of MES to adjust the pH to 3.5, 4.5, 5, 5.5, 6.5, or 7 (three independent experiments). The fluorescence ratio was linear for pH 4.5–7.0.

### Melanin measurements

Melanin from wild type (control), *tpc2*-targeted CRISPR-Cas9 or siRNA-expressing *Oa1*^−/−^ melanocytes was quantified as previously described^32^. The soluble and insoluble fractions of melanocytes were separated after cell lysis with 1% Triton X-100 (Sigma) in PBS pH 7.4. Total protein was measured in the soluble fraction using a BCA Protein Assay Kit (Pierce). The insoluble fraction was dissolved in 1 N NaOH by incubation for 2 h at 85 °C and used to quantify melanin by measuring the optical density of each sample at 405 nm, then fit with a standard curve generated using synthetic melanin (Sigma). Average cellular melanin values were quantified as the ratio between total melanin and total protein (μg melanin/mg protein) from the same dish. Three independent experiments were performed, each in triplicate.

To increase melanin content in B16-F1 melanocytes, mCherry-tagged wild-type or V443I-OCA2 variants were expressed alone or coexpressed with TPC2-GFP for 24 h prior to fixation and imaging and 48 h prior to melanin analysis. Images from three independent experiments were acquired with a laser-scanning confocal microscope (Zeiss LSM 510) using a 63X objective. The melanin from each B16-F1 condition was quantified as described above.

### Data analysis

All data are shown as mean ± s.e.m. Data were considered significant if p < 0.05 using paired or unpaired two-tailed Student *t*-tests or one-way ANOVA.

### Ethical Approval

Bullfrog eyes used for RPE cells were obtained as discarded tissue from Dr. Thomas Roberts’ laboratory at Brown University under protocol #1303990009, approved by the Institutional Animal Care and Use Committee and used in agreement with the approved guidelines and all the ethics rules and regulations.

## Additional Information

**How to cite this article**: Bellono, N. W. *et al.* A melanosomal two-pore sodium channel regulates pigmentation. *Sci. Rep.* **6**, 26570; doi: 10.1038/srep26570 (2016).

## Supplementary Material

Supplementary Information
