# Corrigendum: A melanosomal two-pore sodium channel regulates pigmentation

**DOI:** 10.1038/srep32274

**Published:** 2016-08-30

**Authors:** Nicholas W. Bellono, Iliana E. Escobar, Elena Oancea

Scientific Reports 6: Article number: 2657010.1038/srep26570; published online: 05 27 2016; updated: 08 30 2016

This Article contains an error in Figure 4a: the upper right “hTPC2 rescue” trace is a duplication of the upper left “control” trace. The correct Figure 4 appears below as Figure 1.

## Figures and Tables

**Figure 1:**
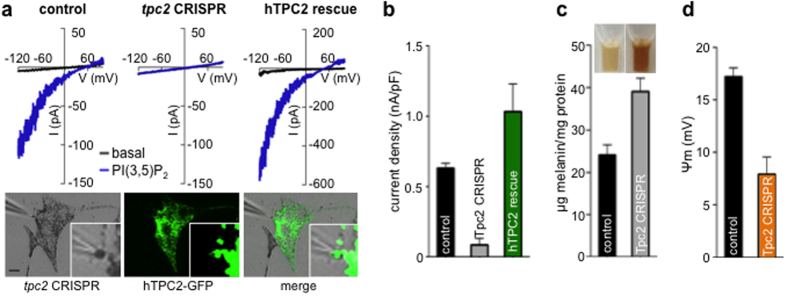
Figure 1.

