# Supplementary material for: A melanosomal two-pore sodium channel regulates pigmentation
Source: Sci Rep. 2016 May 27;6:26570. doi: 10.1038/srep26570 (PMC4882593; doi:10.1038/srep26570)
Supplement: Supplementary Information [file srep26570-s1.pdf]

A melanosomal two-pore sodium channel regulates pigmentation.

Nicholas W. Bellono[#1,2], Iliana E. Escobar[#1], Elena Oancea[1]*

Supplementary Information

Supplementary Figures

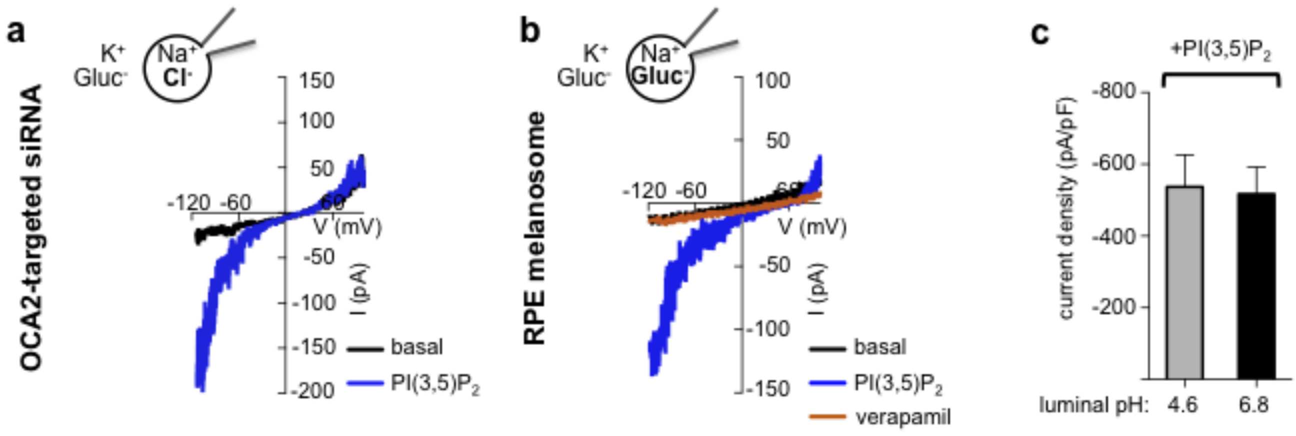

Supplementary Figure 1. I_{PIP2} properties.

a) $PI(3,5)P_2$ activates inwardly rectifying I_{PIP2} in the absence of OCA2-mediated Cl^- currents (representative of n = 2 melanosomes). OCA2 expression was reduced in *Oa1*$^{-/-}$ melanocytes, with OCA2-targeted siRNA[1].

b) In a RPE melanosome, I_{PIP2} is inhibited by 150 µM verapamil, similar to dermal melanosome I_{PIP2} (representative of n = 2 melanosomes).

c) I_{PIP2} current density (pA/pF) measured from *Oa1*$^{-/-}$ melanosomes was similar when the luminal (pipette) pH was 4.6 or 6.8. Average current density (pA/pF) measured at -120 mV (± s.e.m., n = 4 melanosomes).

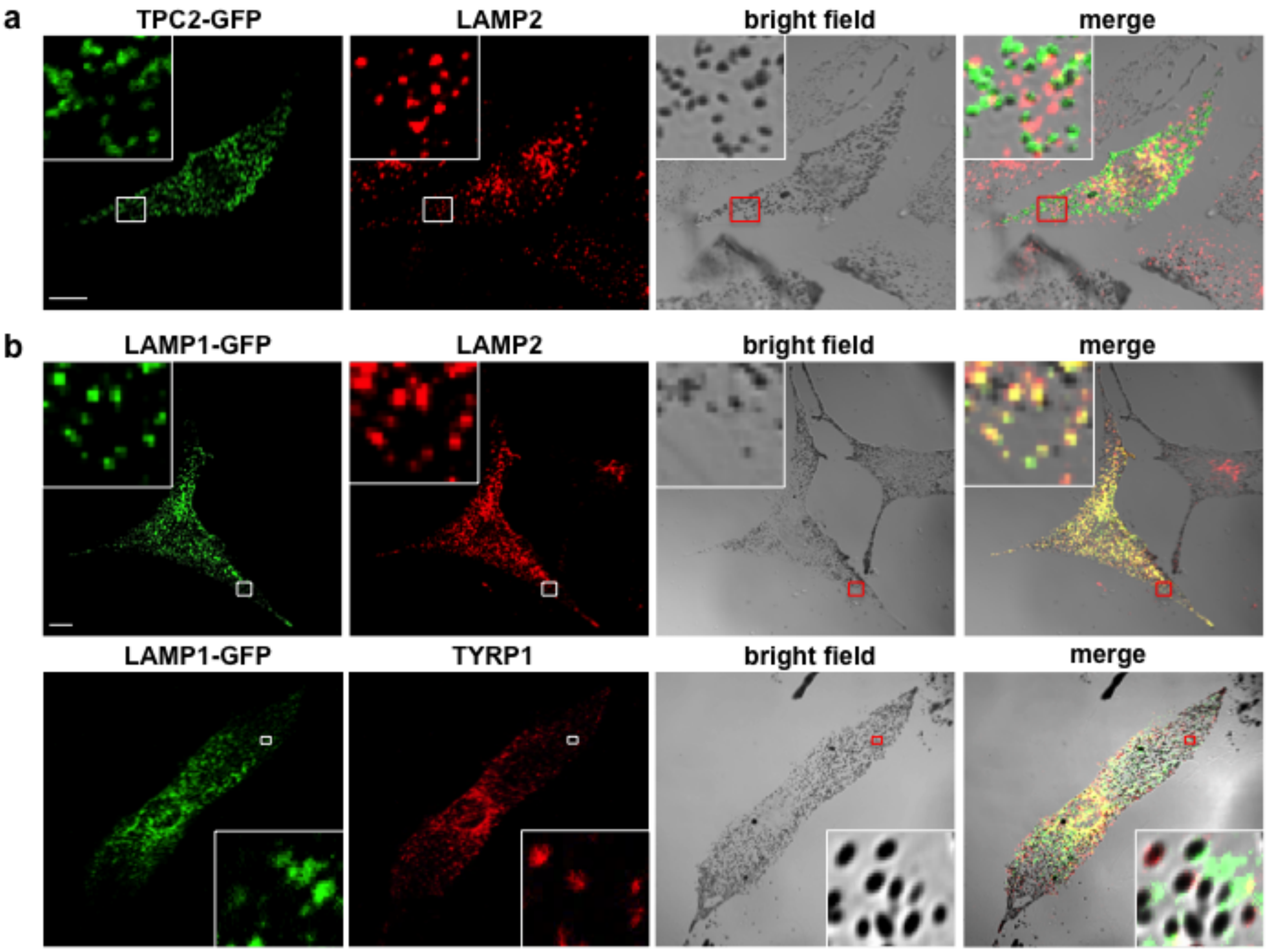

Supplementary Figure 2. TPC2 does not exhibit significant lysosomal localization in pigment cells.

a) In melan-a melanocytes GFP-tagged TPC2 (green) localizes to melanin (50.9 ± 3.2%) (bright field) and TYRP1 (red)-positive compartments, but does not significantly overlap with structures immunolabeled with antibodies against the lysosomal marker LAMP2 (red) (7.0 ± 2.7%, $p < 0.0001$) . Enlarged images of outlined regions shown in lower panels. Scale bar = 10 μm.

b) Expression of the lysosomal protein LAMP1 tagged with GFP (green) localizes to LAMP2 (red)-positive compartments in melan-a cells (71.4 ± 6.2%). LAMP1-GFP (green) does not localize to melanin (bright field) and TYRP1 (red)-positive compartments LAMP1 (12.1 ± 2.1%, $p < 0.0001$).

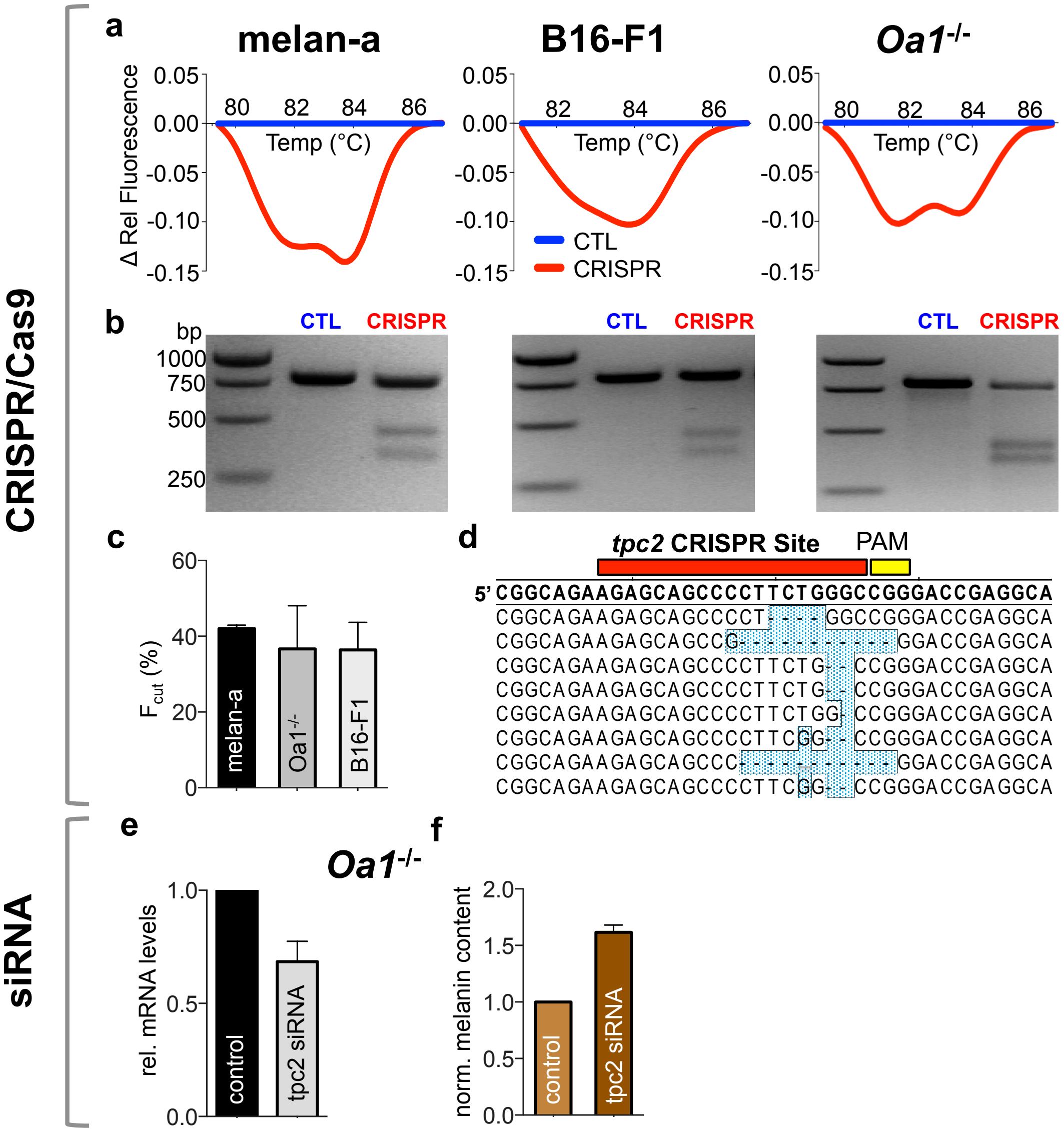

Supplementary Figure 3. Efficiency of *tpc2*-targeted CRISPR-Cas9 and siRNA in melanocytes.

a) High resolution melt analysis of genomic DNA (gDNA) obtained from control (blue) melan-a, B16-F1, or *Oa1*$^{-/-}$ melanocytes and *tpc2*-targeted CRISPR-Cas9 (red) populations of the same types of cells. Differences in the relative fluorescence intensity (Δ Rel Fluorescence) between the control and CRISPR-Cas9 treated cells are due to gDNA mutations in the targeted sequence.

b) Representative gels from mutation detection assay using gDNA from melan-a, B16-F1, or *Oa1*$^{-/-}$ melanocytes expressing control or *tpc2*-targeted CRISPR-Cas9. Cleaved DNA fragments are due to indels caused by CRISPR-Cas9-induced mutations.

c) Average fraction of cleaved fragments (F_{cut}) from Guide-it Resolvase assay determined from n = 3 independent experiments.

d) Identification of individual mutations in melanocytes treated with *tpc2*-targeted CRISPR-Cas9 using single gDNA species cloned in the TOPO vector. The sequences from each clone were aligned with the wild-type sequence (in bold) revealing a range of deletions and insertions the *tpc2* gene (highlighted in blue) at the CRISPR site.

e) Mouse tpc2-targeted siRNA stably expressed in *Oa1*$^{-/-}$ melanocytes reduced the TPC2 mRNA levels by ~30%, compared to control siRNA. (± s.e.m., $n = 3$, $p < 0.01$)

f) Melanocytes expressing TPC2-targeted siRNA have ~50% higher melanin content than control siRNA expressing cells. (± s.e.m., $n = 3$, $p < 0.001$)

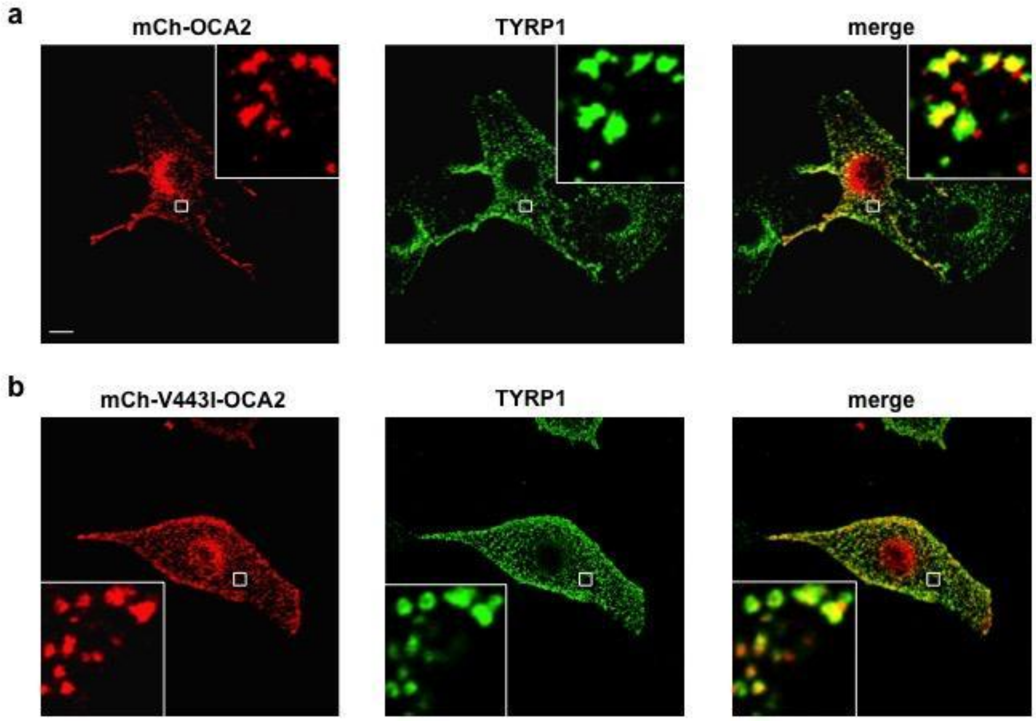

Supplementary Figure 4. WT and V443I OCA2 variants localize to melanosomes in B16-F1 melanocytes.

a) In B16-F1 melanocytes mCherry-tagged wild type (WT) OCA2 (red) localizes to TYRP1 immunostained (green) compartments. Enlarged images of outlined regions shown in lower panels. Scale bar = 10 μm.

b) mCherry-tagged V443I mutant OCA2 (red) localizes to TYRP1 immunostained (green) compartments in B16-F1 cells.

Supplementary reference

1 Bellono, N. W., Escobar, I. E., Lefkovith, A. J., Marks, M. S. & Oancea, E. An intracellular anion channel critical for pigmentation. *eLife* **3** (2014).
